# Supplementary material for: The myokine Fibcd1 is an endogenous determinant of myofiber size and mitigates cancer-induced myofiber atrophy
Source: Nat Commun. 2022 May 2;13:2370. doi: 10.1038/s41467-022-30120-1 (PMC9061726; doi:10.1038/s41467-022-30120-1)
Supplement: Supplementary file 1 — Supplementary Information [file 41467_2022_30120_MOESM1_ESM.pdf]

SUPPLEMENTARY INFORMATION

The myokine Fibcd1 is an endogenous determinant of myofiber size and mitigates cancer-induced myofiber atrophy

Flavia A. Graca, Mamta Rai, Liam C. Hunt, Anna Stephan, Yong-Dong Wang, Brittney Gordon, Ruishan Wang, Giovanni Quarato, Beisi Xu, Yiping Fan, Myriam Labelle, Fabio Demontis

SUPPLEMENTARY FIGURES AND FIGURE LEGENDS

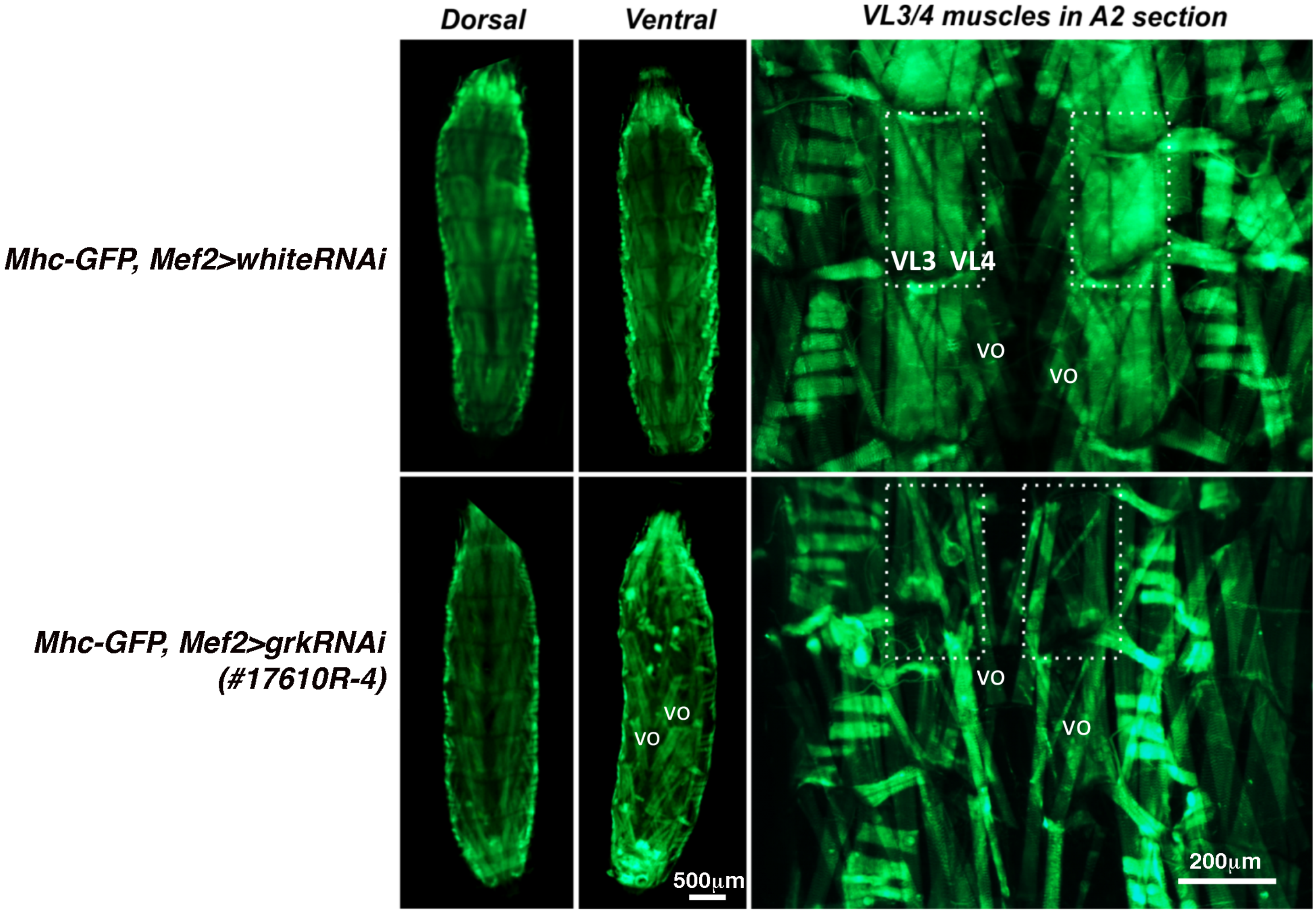

Supplementary Fig. 1. RNAi for the EGF-like ligand gurken leads to type-specific muscle degeneration in *Drosophila* larvae. Representative images of *Drosophila* larvae (dorsal and ventral views) and dissected body wall skeletal muscles that express a GFP-tagged version of myosin heavy chain (Mhc-GFP). RNAi is driven by *Mef2-Gal4*, which drives transgene expression in all body wall skeletal muscles. An extensive characterization of the *Mef2-Gal4* driver is available in ref.1[1]. RNAi for the EGF-like ligand gurken (grk), previously implicated in dorsoventral patterning in the *Drosophila* egg and embryo[2], leads to selective muscle degeneration. Specifically, ventral lateral muscles (VL) degenerate in *Mef2>grk*^{RNAi} larvae, whether they are present as expected in control *Mef2>white*^{RNAi} larvae. However, other muscles such as ventral oblique (VO) muscles are present, indicating that grk/EGF-like signaling is not necessary or required at lower levels for their differentiation and growth. These findings indicate a role for myokines in developmental patterning and differentiation of specific skeletal muscles.

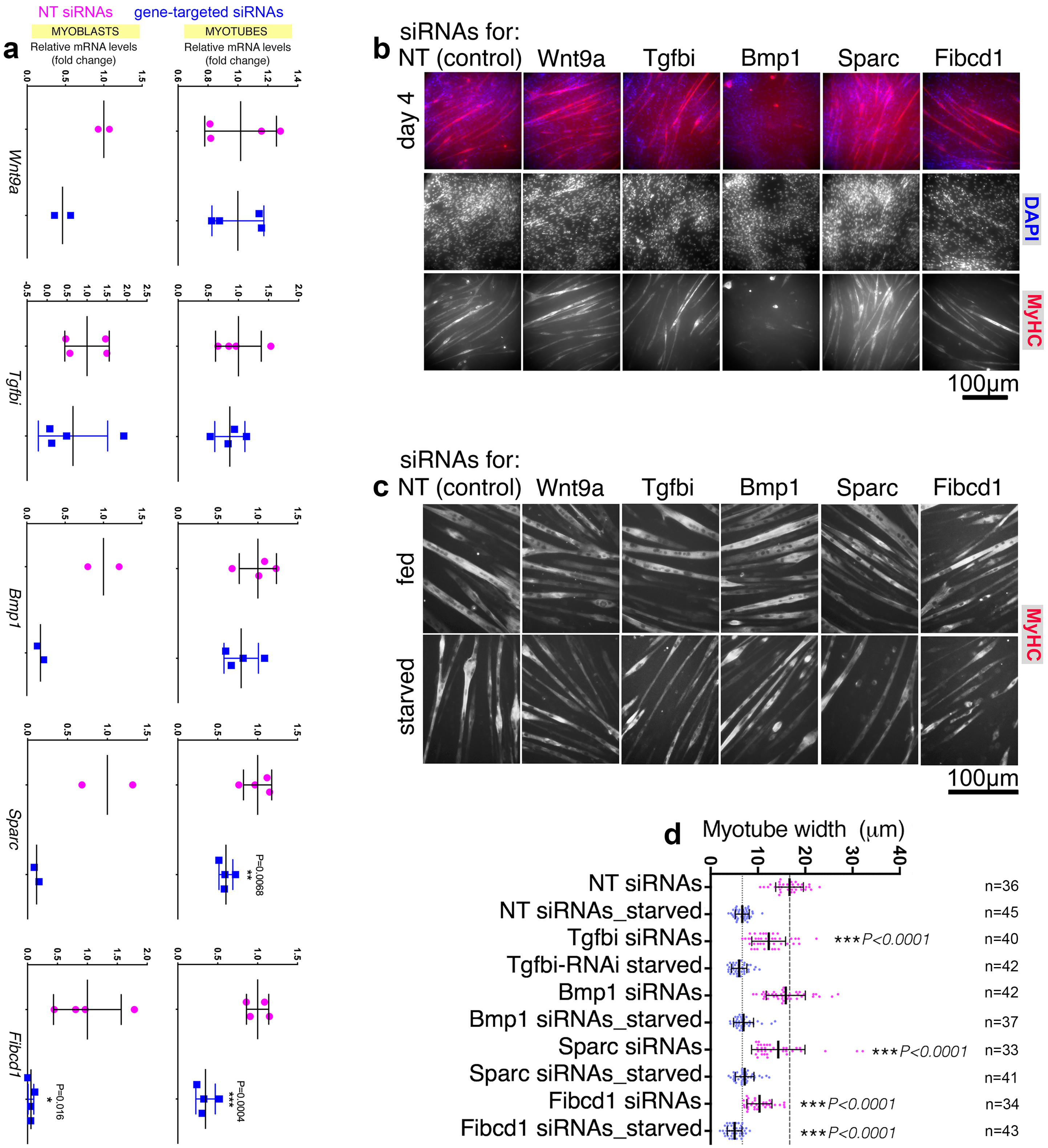

Supplementary Fig. 2. Testing myokines orthologous to *Drosophila* screen hits for their capacity to modulate mouse C2C12 myoblast fusion and myotube size. a, qRT-PCR-based validation of siRNA-mediated knockdown of target myokines in mouse C2C12 myoblasts and myotubes, compared to control non-targeting (NT) siRNAs. Significant knockdown is found for Sparc and Fibcd1. Mean values are shown with n=2 (myoblasts), n=4 (myotubes), and n=4 (Fibcd1 siRNAs,

both myoblasts and myotubes) biologically independent samples; error bars indicate the SD; *$P<0.05$, **$P<0.01$, and ***$P<0.001$. *P* values were determined by two-tailed unpaired t-tests.
b, Effect of siRNAs targeting myokines on C2C12 myoblast fusion. Immunostaining of cultured C2C12 myotubes with the pan-myosin heavy chain (MyHC; #MF20) antibody at day 4 after the induction of differentiation. Note that Bmp1 siRNAs reduce myoblast fusion whereas this is not substantially affected by siRNAs for Sparc and Fibcd1.
c-d, Effect of siRNAs targeting myokines on C2C12 myotube size. siRNA treatment of myoblast-depleted cultures of C2C12 myotubes indicates that siRNAs for Tgfbi, Sparc, and Fibcd1 significantly reduces myotube size, in comparison to control NT siRNAs. Note that Fibcd1 siRNAs induce the strongest decrease in myofiber size. Mean values ± SD are shown; ***$P<0.001$. The n of biologically independent samples (myotubes) is reported in the figure. *P* values were determined by two-way ANOVA with Sidak's multiple comparisons test.
Source data is provided in the Source Data file.

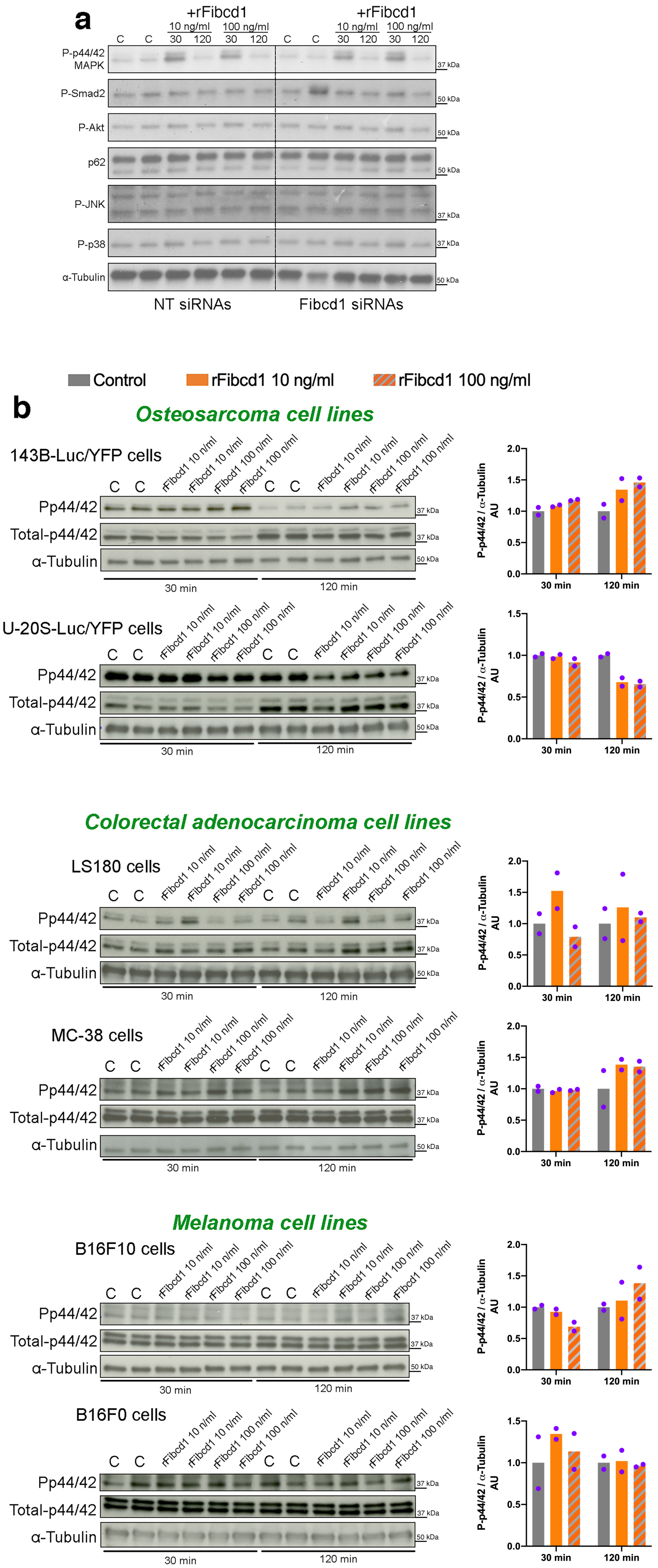
a
+rFibcd1
10 ng/ml
100 ng/ml
C
30
120
P-p44/42 MAPK
P-Smad2
P-Akt
p62
P-JNK
P-p38
α-Tubulin
37 kDa
50 kDa
NT siRNAs
Fibcd1 siRNAs
Control
rFibcd1 10 ng/ml
rFibcd1 100 ng/ml
b
Osteosarcoma cell lines
143B-Luc/YFP cells
U-20S-Luc/YFP cells
Colorectal adenocarcinoma cell lines
LS180 cells
MC-38 cells
Melanoma cell lines
B16F10 cells
B16F0 cells
rFibcd1 10 n/ml
rFibcd1 100 n/ml
Pp44/42
Total-p44/42
α-Tubulin
30 min
120 min
P-p44/42 / α-Tubulin AU
0.0
0.5
1.0
1.5
2.0

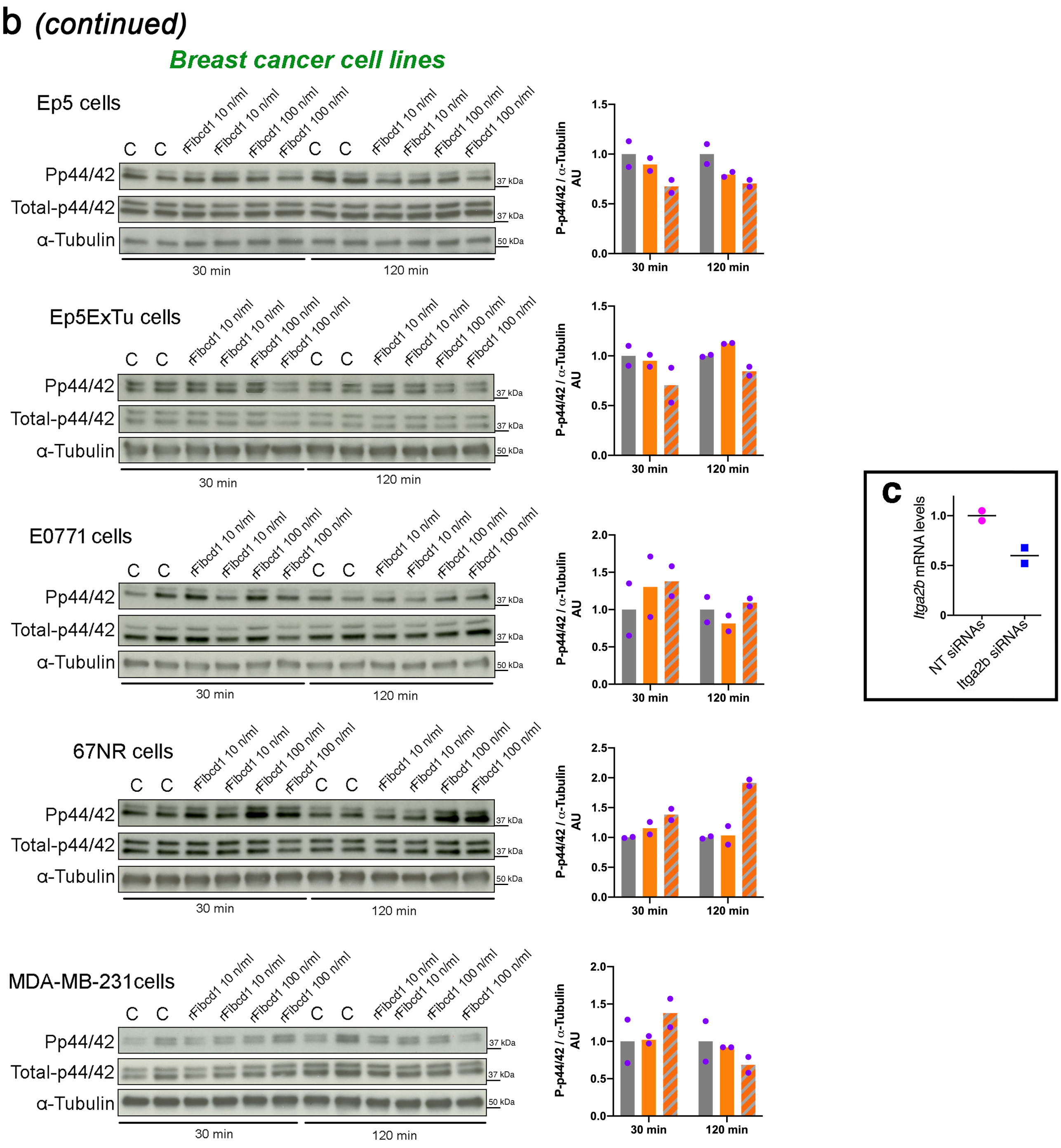

Supplementary Fig. 3. Modulation of P-p42/44 MAPK by rFibcd1. a, Treatment of C2C12 myotubes with recombinant Fibcd1 (rFibcd1) at 10 and 100 ng/mL for 30 or 120 minutes. rFibcd1 promotes ERK/MAPK signaling, as indicated by the increase in p42/44 phosphorylation following rFibcd1 treatment, compared to control (C). rFibcd1 does not impact the levels of other myofiber size regulators (P-Smad2, P-Akt, P-Jnk, and P-p38), and of the autophagic substrate p62.
b, Treatment with rFibcd1 marginally impacts P-p42/44 MAPK levels in most cancer cell lines tested with the exception of 143B-Luc/YFP osteosarcoma and 67NR breast cancer cells, in which rFibcd1 increases P-p42/44 levels, indicative of ERK/MAPK signaling (mean values are shown with n=2 biologically independent samples).
c, siRNAs for Itga2b reduce Itga2b mRNA levels in C2C12 myotubes, compared to control NT siRNAs (mean values are shown with n=2 RNA preps from independent cell cultures and siRNA treatments). Source data is provided in the Source Data file.

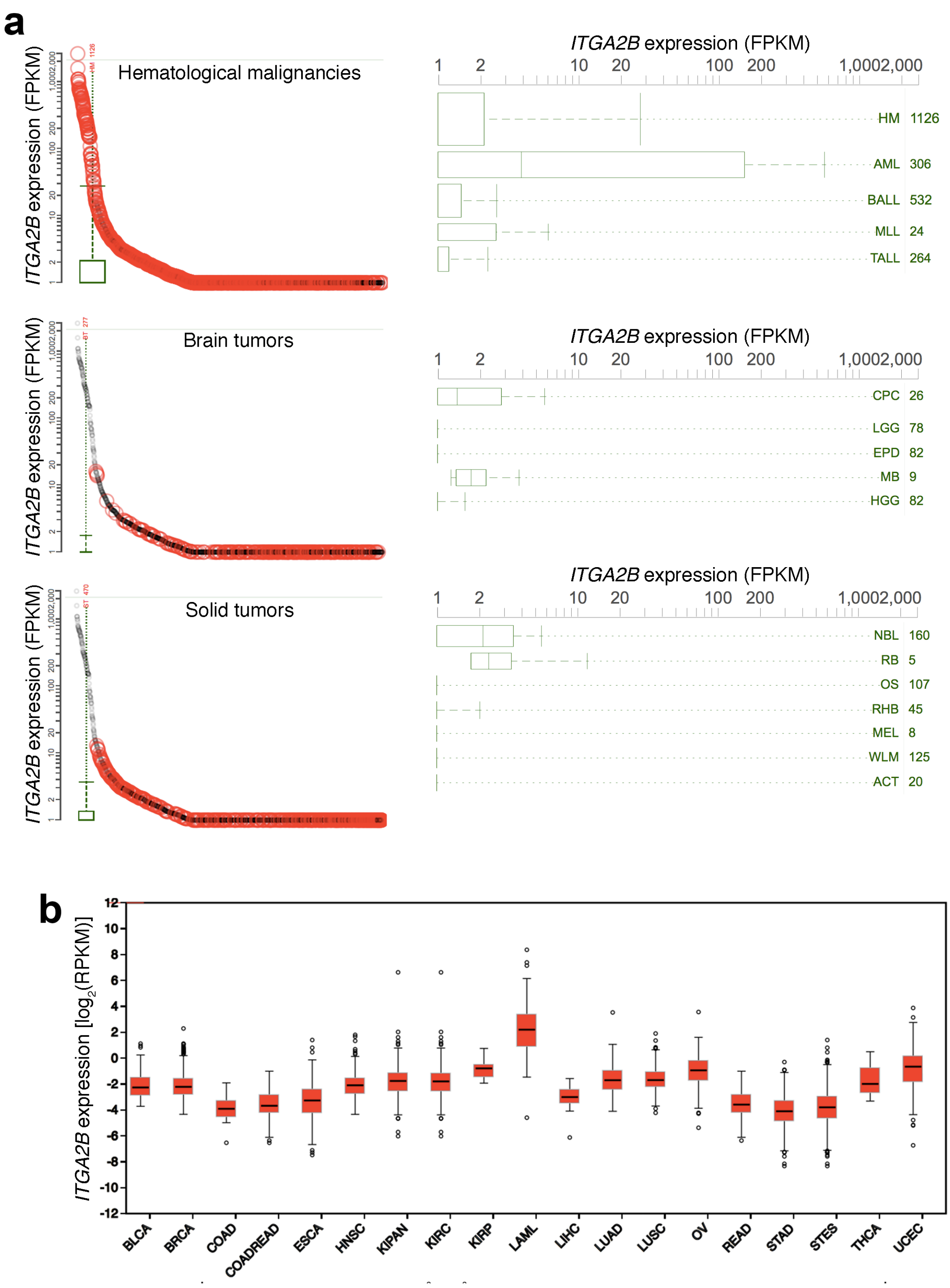

Supplementary Fig. 4. *ITGA2B* is poorly expressed in pediatric and adult cancers. a-b, RNA-seq data from pediatric cancers (**a**; from: https://proteinpaint.stjude.org/) and adult cancers (**b**; from http://firebrowse.org/viewGene.html?gene=ITGA2B) indicates that most cancers display little (if any) *ITGA2B* expression. In **a-b**, each box plot represents the 1st quartile, median, and 3rd quartile. In **a**, the n of tumor samples is reported in the figure. In **b**, the n of tumor samples is the following: BLCA (n=56), BRCA (n=778), COAD (n=10), COADREAD (n=81), ESCA (n=185), HNSC (n=263), KIPAN (n=483), KIRC (n=469), KIRP (n=14), LAML (n=179), LIHC (n=17), LUAD (n=125), LUSC (n=223), OV (n=299), READ (n=71), STAD (n=273), STES (n=458), THCA (n=3), and UCEC (n=266).

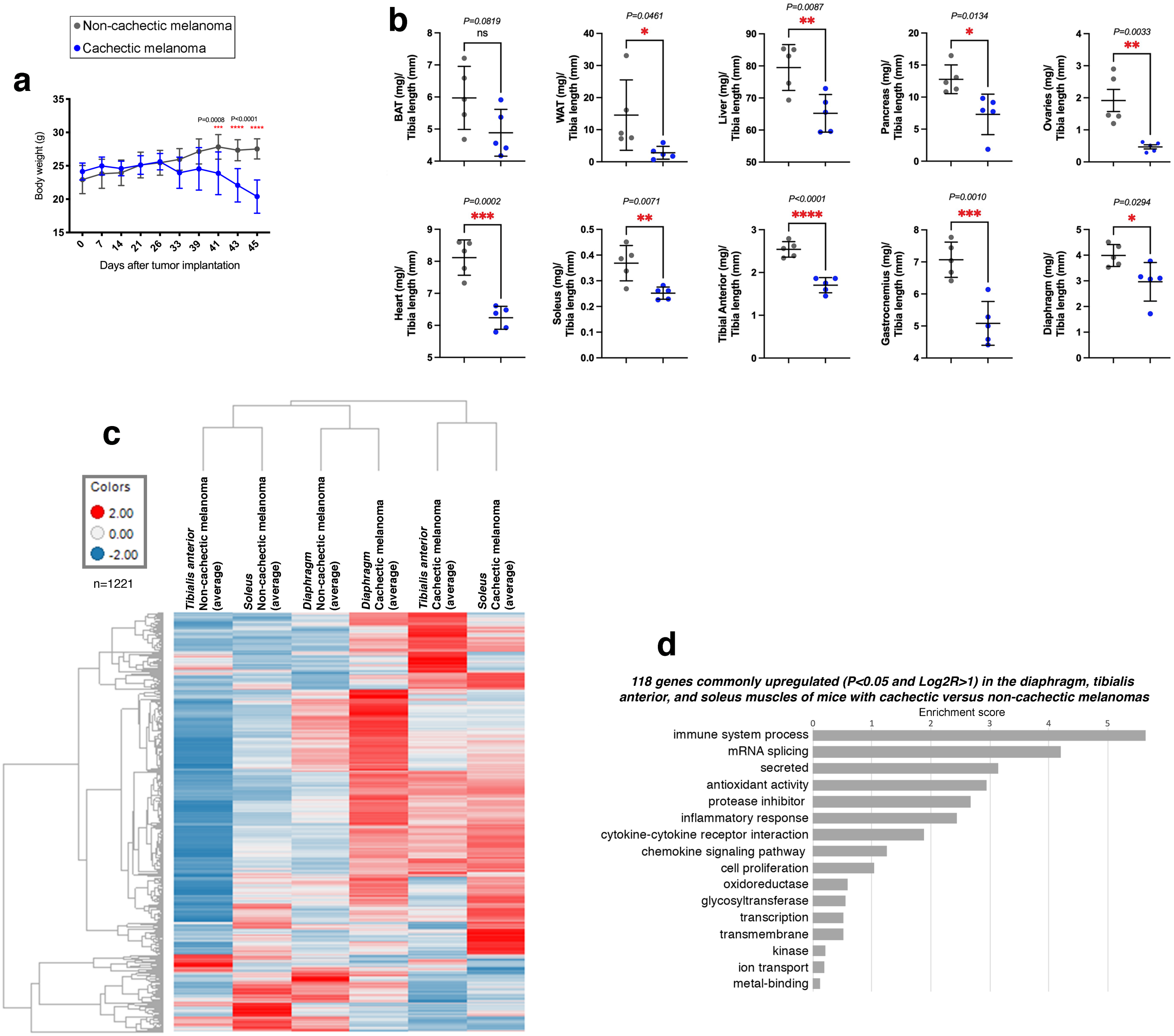

Supplementary Fig. 5. Characterization of cachexia induced by orthotopic patient-derived melanoma xenografts.

a, Body weight decreases by ~25% in ~7 weeks in response to the subcutaneous injection of a cachexia-inducing ("cachectic") melanoma (MAST360B/SJMEL030083_X2; n=7 biologically independent mice) into 2-month-old mice compared to a control cachexia-non-inducing ("non-cachectic") melanoma (MAST552A/SJMEL031086_X1; n=10 biologically independent mice) which does not lead to body weight loss. Mean values ± SD are indicated with ****P*<0.001. *P* values were determined by two-way ANOVA with Tukey's multiple comparisons test.

b, Impact of melanoma-induced cachexia on the weight of different tissues. The weight of the gonadal white adipose tissue (WAT), liver, pancreas, ovaries, heart, and skeletal muscles (soleus, tibialis anterior, gastrocnemius, and diaphragm) is significantly reduced at day 42 post tumor cell injection in response to implantation of the cachectic melanoma compared with the control non-cachectic melanoma, whereas the weight of the interscapular brown adipose tissue (BAT) does not significantly change. Tissue/organ weight is normalized to the tibia length. Mean values ± SD are indicated with n=5

biologically independent samples (tissues/organs obtained from independent mice) and *$P<0.05$; **$P<0.01$; ***$P<0.001$. *P* values were determined by two-tailed unpaired t-tests.

c, Heatmap that displays the expression of 1221 genes as detected from RNA-sequencing of diaphragm, tibialis anterior, and soleus muscles from mice with cachectic versus non-cachectic melanomas; n=5 biologically independent samples (tibialis anterior and soleus muscles, each sourced from an independent mouse), and n=9 biologically independent samples (diaphragm muscles, each obtained from an independent mouse). Common and muscle-specific changes are induced. The heatmaps are based on z-scores of group averages from baseline-adjusted log2(TMP) for genes with at least one significant call among the related comparison sets.

d, GO terms describing 118 genes that are commonly upregulated in the diaphragm, tibialis anterior, and soleus muscles of mice with cachectic (MAST360B/SJMEL030083_X2) versus non-cachectic (MAST552A/SJMEL031086_X1) melanomas, with $P<0.05$ and Log2R>1 (n=5/group for the tibialis anterior and soleus muscles, and n=9 diaphragm muscles). Only 8 genes are commonly downregulated. Supplementary Data 4 reports the full set of commonly upregulated and downregulated genes.

Source data is provided in the Source Data file.

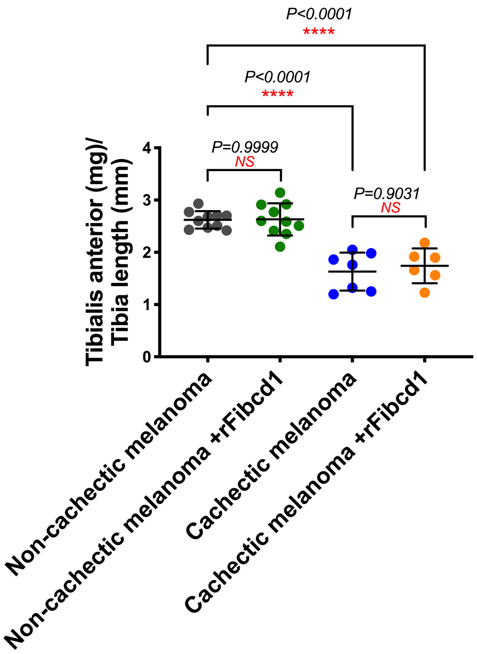

Supplementary Fig. 6. Intraperitoneal injection of rFibcd1 does not impact cancer-induced wasting of the tibialis anterior muscle. Tibialis anterior (TA) muscle mass normalized to tibia length. Cancer-induced cachexia significantly reduces the weight of the TA muscle, located in the hindleg. However, intraperitoneal injection of rFibcd1 does not significantly impact TA mass. Mean ± SD is indicated with n=10 for non-cachectic melanomas, n=7 for cachectic melanomas, and n=6 for cachectic melanomas treated with rFibcd1; each n corresponds to a biologically independent sample (tibialis anterior muscle) obtained from an independent mouse; NS= not significant; ****P<0.0001. P values were determined by one-way ANOVA with Tukey's multiple comparisons test.
Source data is provided in the Source Data file.

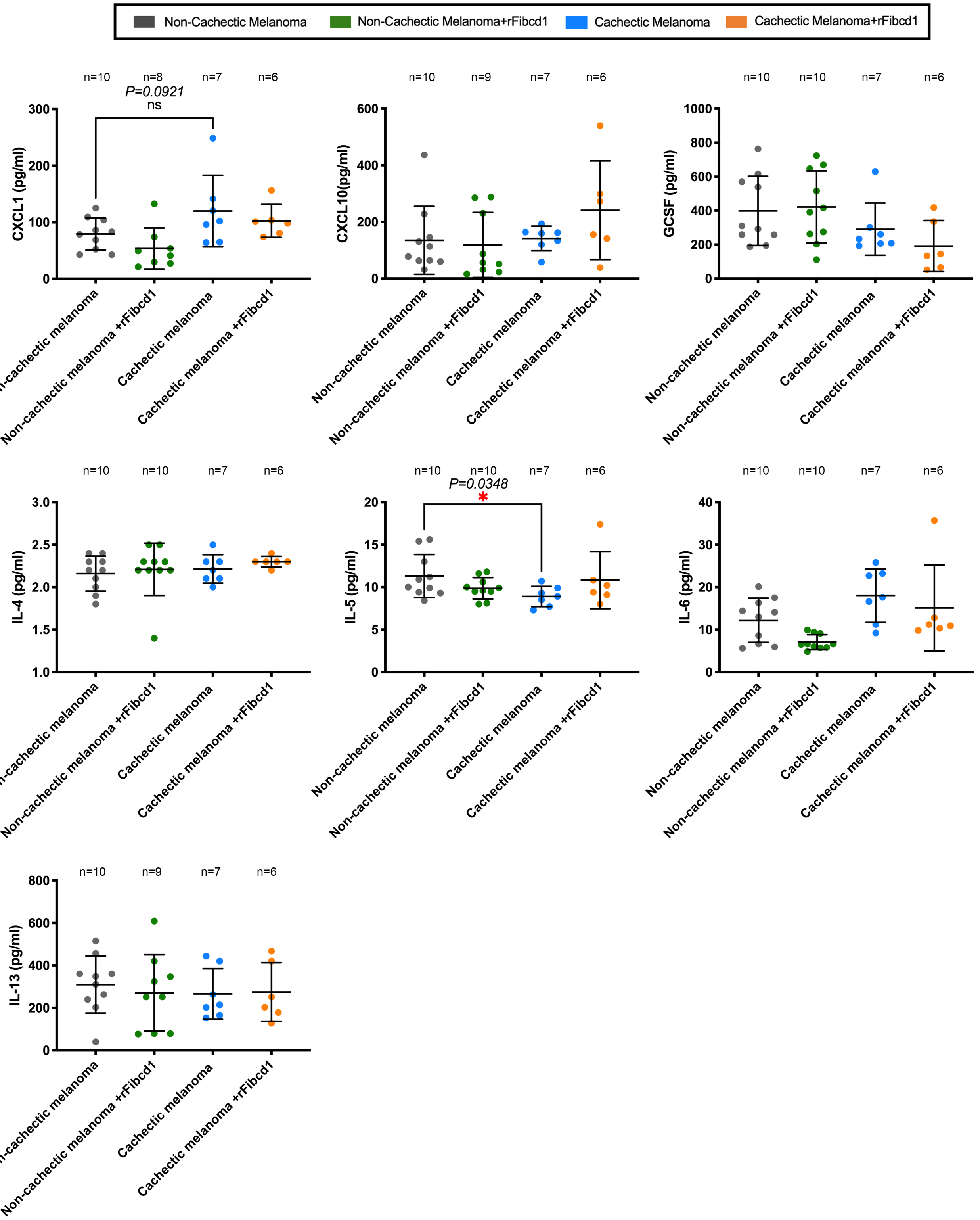

Supplementary Fig. 7. Levels of inflammatory cytokines in the serum of mice with melanoma xenografts and with or without rFibcd1 treatments.

The levels of most inflammatory cytokines tested are not significantly regulated by cachectic versus non-cachectic melanoma xenografts and by rFibcd1 versus mock treatments at end point. Mean values ± SD are indicated with biologically independent plasma samples obtained from independent mice (the precise number is indicated in the figure); **P*<0.05. *P* values were determined by one-way ANOVA with Tukey's multiple comparisons test. Source data is provided in the Source Data file.

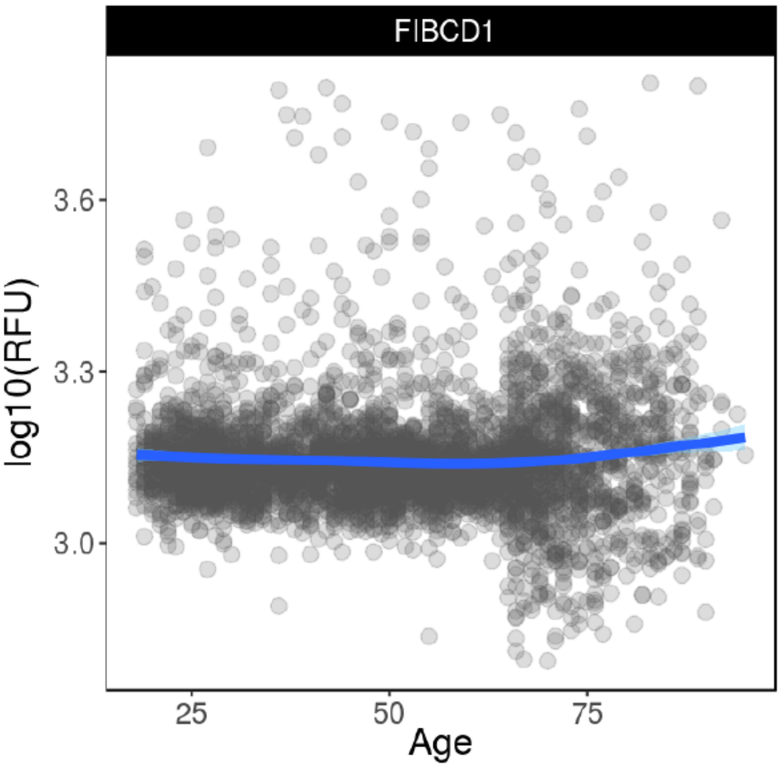

ID	FIBCD1.9378.6.3
p.Age	0.504755923
q.Age	0.615684352
p.Sex	0.271845578
q.Sex	0.349669444

Supplementary Fig. 8. FIBCD1 is detected in the human plasma. FIBCD1 is detected in the human plasma and its levels do not change with aging. This data was retrieved from the Aging Plasma Proteome dataset[3] (https://twc-stanford.shinyapps.io/aging_plasma_proteome/).

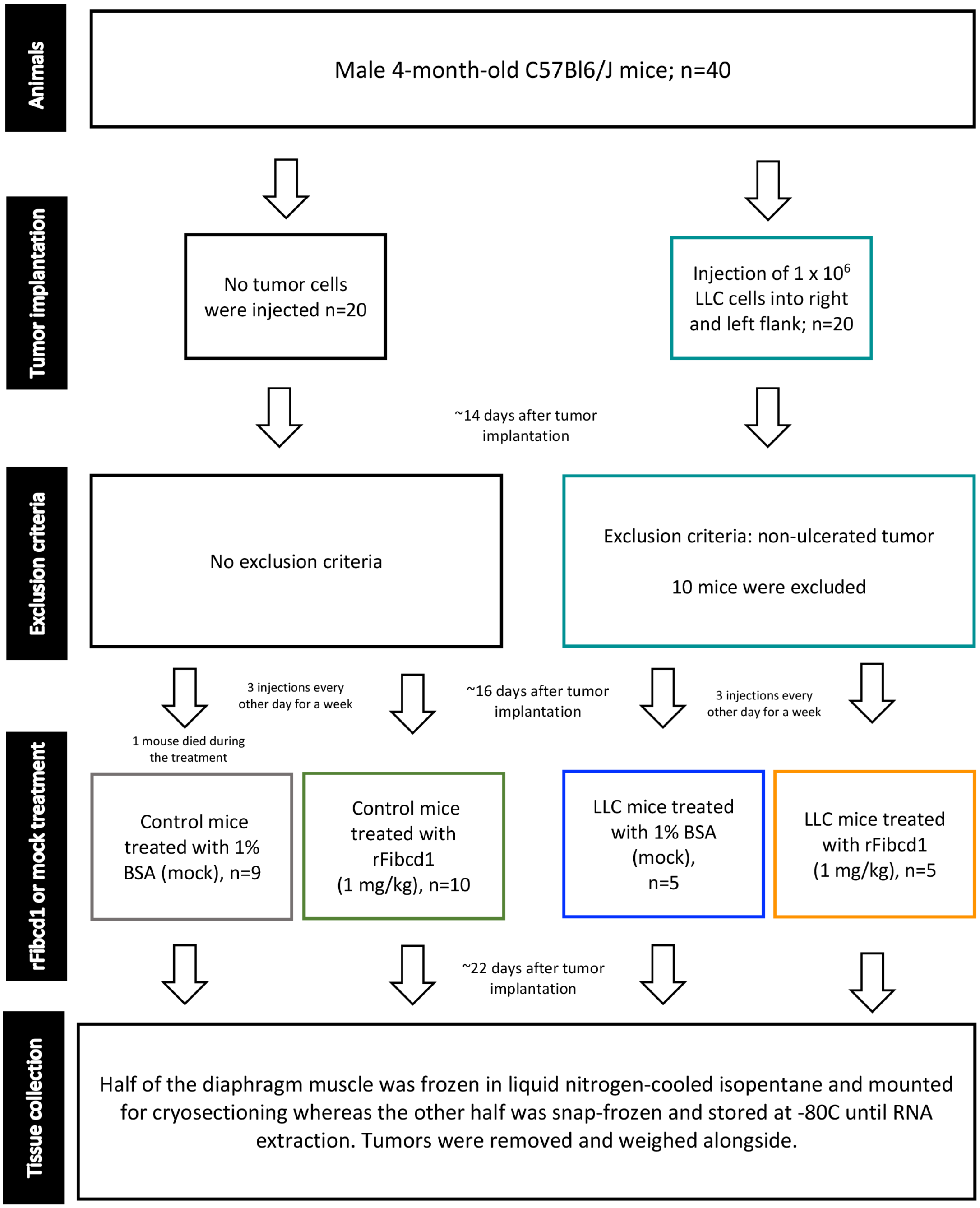

Supplementary Fig. 9. Flow diagram of studies with mice implanted with LLC cancer cells. Mice were randomly allocated into treatment groups (mock versus rFibcd1). However, 10 mice were excluded because they carried non-ulcerated tumors, which develop minimal cachexia. A mouse in the control group not implanted with LLC cancer cells was excluded as it died during treatment. An investigator blinded to the experimental conditions assessed the result of rFibcd1 treatment.

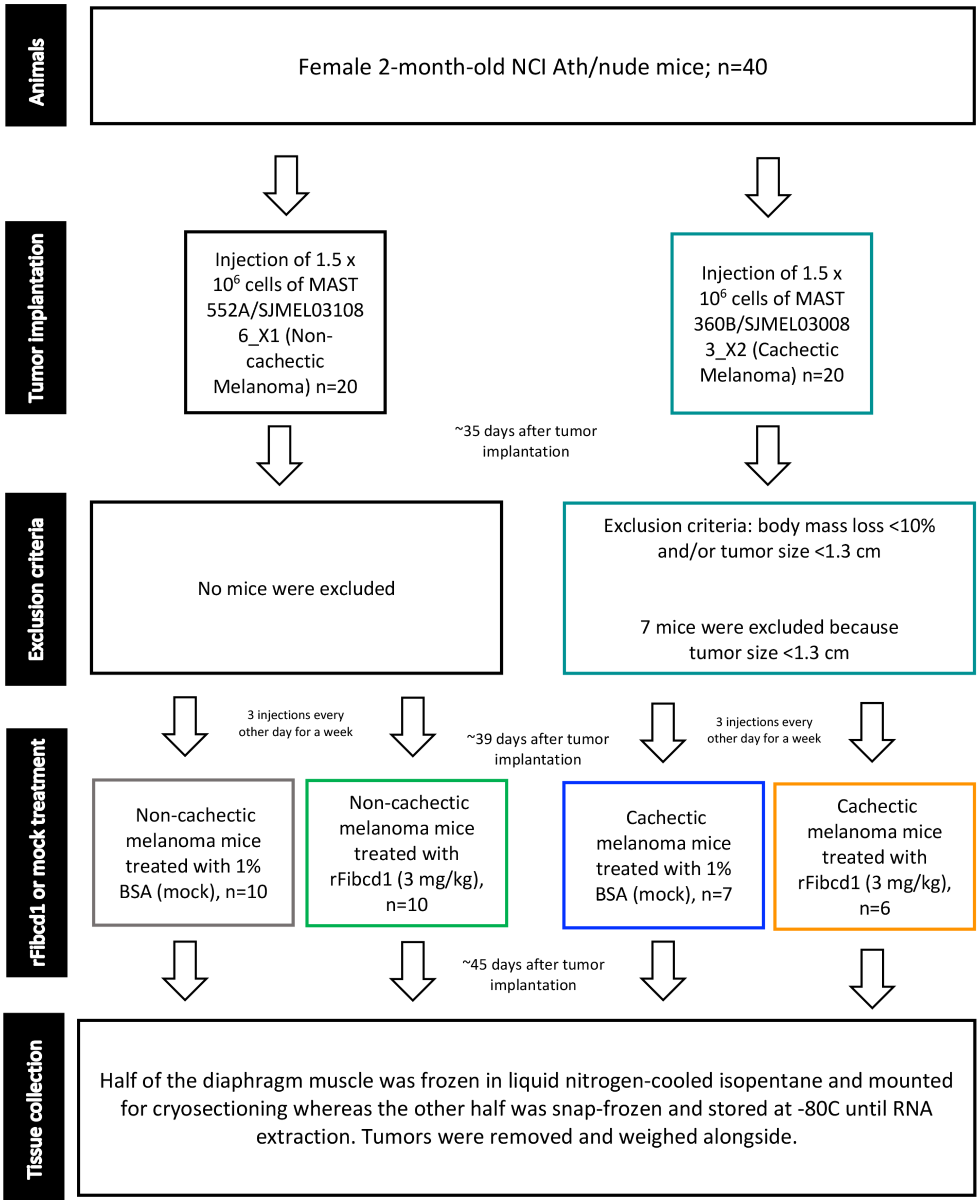

Supplementary Fig. 10. Flow diagram of studies with mice implanted with cachectic and non-cachectic melanoma xenografts. Mice were randomly allocated into treatment groups (mock versus rFibcd1). However, 7 mice with cachectic melanoma xenografts were excluded from treatment because they developed minimal cachexia, as indicated by a decline in body weight of less than 10% and/or limited tumor burden (<1.3 cm tumor diameter). An investigator blinded to the experimental conditions assessed the result of rFibcd1 treatment.

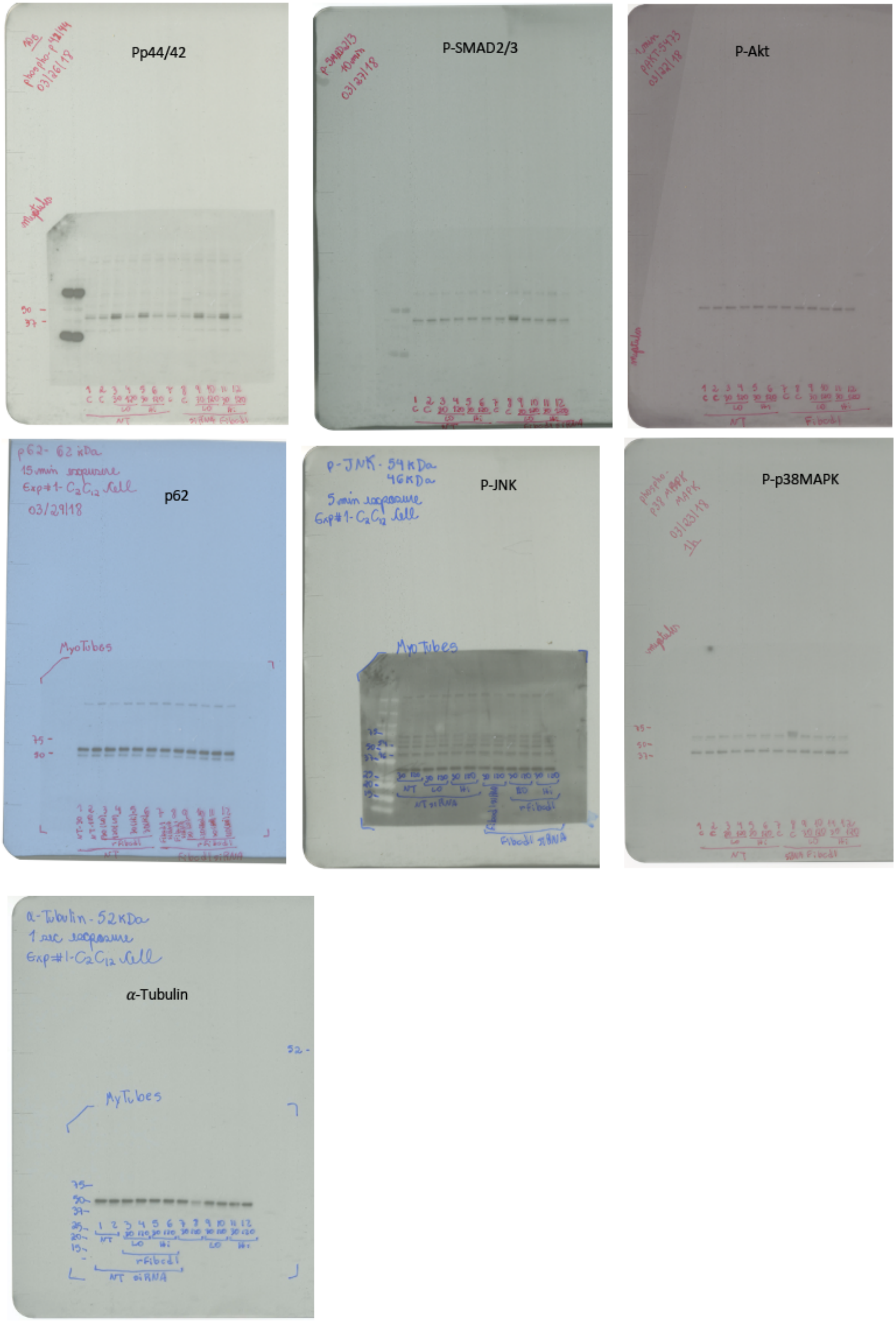

Full scans of blots shown in Supplementary Fig. 3a

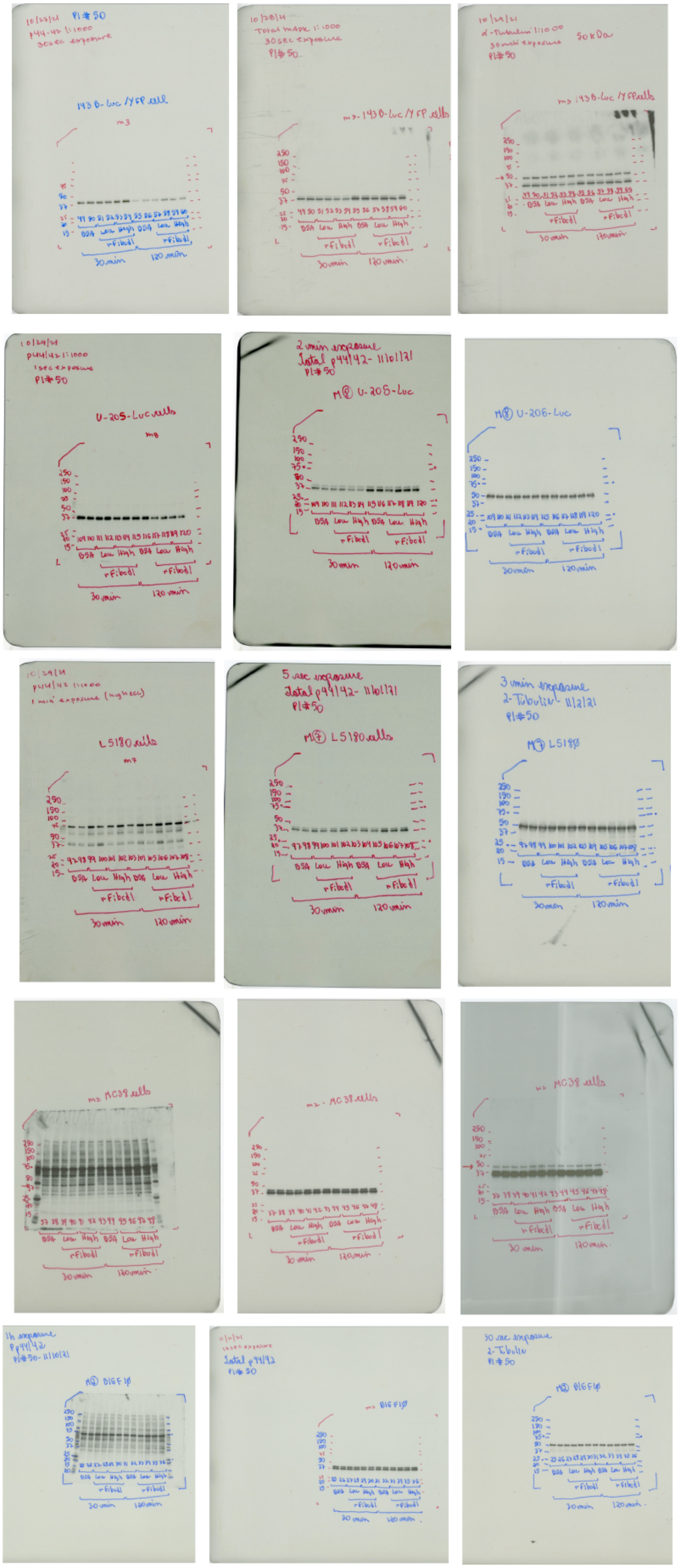

Full scans of blots shown in Supplementary Fig. 3b (part 1)

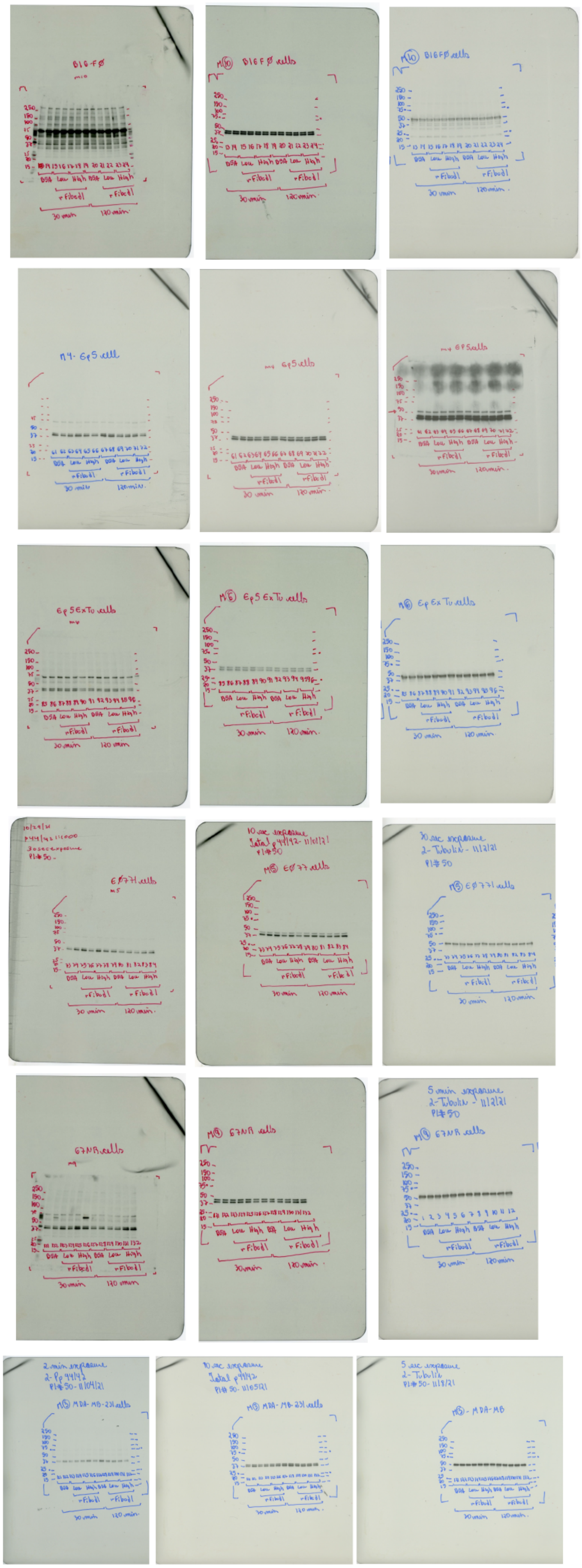

Full scans of blots shown in Supplementary Fig. 3b (part 2)

SUPPLEMENTARY DATA

Supplementary Data 1. *Drosophila* RNAi screening identifies evolutionary conserved myokines that regulate myofiber size.

Supplementary Data 2. Genes regulated in diaphragm muscles from mice with LLC cancers but not in diaphragm muscles from mice with LLC cancers upon treatment with rFibcd1.

Supplementary Data 3. qRT-PCR oligos.

Supplementary Data 4. Genes that are commonly upregulated and downregulated in the diaphragm, tibialis anterior, and soleus muscles of mice with cachectic versus non-cachectic melanomas.

Supplementary Data 5. Genes differentially regulated in diaphragm muscles of mice with cachectic versus non cachectic melanomas, and treated with either rFibcd1 or mock.

Source Data File. Additional primary data and full scans of blots.

SUPPLEMENTARY REFERENCES

1 Demontis, F. & Perrimon, N. Integration of Insulin receptor/Foxo signaling and dMyc activity during muscle growth regulates body size in Drosophila. *Development* **136**, 983-993, doi:10.1242/dev.027466 (2009).
2 Schupbach, T. & Roth, S. Dorsoventral patterning in Drosophila oogenesis. *Curr Opin Genet Dev* **4**, 502-507, doi:10.1016/0959-437x(94)90064-a (1994).
3 Lehallier, B. *et al.* Undulating changes in human plasma proteome profiles across the lifespan. *Nat Med* **25**, 1843-1850, doi:10.1038/s41591-019-0673-2 (2019).
