# Supplementary material for: The myokine Fibcd1 is an endogenous determinant of myofiber size and mitigates cancer-induced myofiber atrophy
Source: Nat Commun. 2022 May 2;13:2370. doi: 10.1038/s41467-022-30120-1 (PMC9061726; doi:10.1038/s41467-022-30120-1)
Supplement: Supplementary file 2 — Reporting Summary [file 41467_2022_30120_MOESM2_ESM.pdf]

nature research

Corresponding author(s): Fabio Demontis

Last updated by author(s): 28 February 2022

# Reporting Summary

Nature Research wishes to improve the reproducibility of the work that we publish. This form provides structure for consistency and transparency in reporting. For further information on Nature Research policies, see our Editorial Policies and the Editorial Policy Checklist.

## Statistics

For all statistical analyses, confirm that the following items are present in the figure legend, table legend, main text, or Methods section.

| n/a | Confirmed | |
|---|---|---|
| ☐ | ☒ | The exact sample size ($n$) for each experimental group/condition, given as a discrete number and unit of measurement |
| ☐ | ☒ | A statement on whether measurements were taken from distinct samples or whether the same sample was measured repeatedly |
| ☐ | ☒ | The statistical test(s) used AND whether they are one- or two-sided<br>*Only common tests should be described solely by name; describe more complex techniques in the Methods section.* |
| ☐ | ☒ | A description of all covariates tested |
| ☐ | ☒ | A description of any assumptions or corrections, such as tests of normality and adjustment for multiple comparisons |
| ☐ | ☒ | A full description of the statistical parameters including central tendency (e.g. means) or other basic estimates (e.g. regression coefficient) AND variation (e.g. standard deviation) or associated estimates of uncertainty (e.g. confidence intervals) |
| ☐ | ☒ | For null hypothesis testing, the test statistic (e.g. $F$, $t$, $r$) with confidence intervals, effect sizes, degrees of freedom and $P$ value noted<br>*Give P values as exact values whenever suitable.* |
| ☒ | ☐ | For Bayesian analysis, information on the choice of priors and Markov chain Monte Carlo settings |
| ☒ | ☐ | For hierarchical and complex designs, identification of the appropriate level for tests and full reporting of outcomes |
| ☒ | ☐ | Estimates of effect sizes (e.g. Cohen's $d$, Pearson's $r$), indicating how they were calculated |

*Our web collection on statistics for biologists contains articles on many of the points above.*

## Software and code

Policy information about availability of computer code

| | |
|---|---|
| Data collection | Data organization, scientific graphing and statistical analyses were performed with Microsoft Excel (version 14.7.3) and GraphPad Prism (version 6) |
| Data analysis | RNA-seq: RNA sequencing libraries for each sample were prepared with 1 mg total RNA by using the Illumina TruSeq RNA Sample Prep v2 Kit per the manufacturer's instructions, and sequencing was completed on the Illumina NovaSeq 6000. The 100-bp paired-end reads were trimmed, filtered against quality (Phred-like Q20 or greater) and length (50-bp or longer), and aligned to a mouse reference sequence GRCm38 (UCSC mm10) by using CLC Genomics Workbench v12.0.1 (Qiagen). For gene expression comparisons, we obtained the TPM (transcript per million) counts from the RNA-Seq Analysis tool. The differential gene expression analysis was performed by using the non-parametric ANOVA using the Kruskal-Wallis and Dunn's tests on log-transformed TPM values between biological replicates of experimental groups, implemented in Partek Genomics Suite v7.0 software (Partek Inc.).<br>The average TPM counts from each experimental group for significant genes (defined as indicated in the corresponding figure legends) were further clustered in a heatmap using z-score normalization and similarity measure by correlation. Specifically, for the heatmap in Fig. 4, the cutoff for significance is p<0.05 and log2R>0.585 (50% change) between these sets, and genes with significant call for at least one of the comparisons were included in the heatmap. The heatmaps in Fig. 5 includes all genes with significance in at least one of eight comparisons, with cutoffs of significance at p<0.01 and log2R>1. The heatmap in Supplementary Fig. 5 was generated following the same criteria as for Fig. 5. For the heatmap in Fig.7, the cutoff of significance was set at p<0.05 with log2R>1 (one-fold change) for the comparison of CAC versus NCAC, and p<0.05 with log2R>0.585 (50% change) for the comparison of rFibcd1 versus mock treatment.<br>The RNA-seq data discussed in this publication has been deposited in the NCBI's Gene Expression Omnibus and is accessible through GEO Series accession numbers GSE156815, GSE158581, and GSE183833.<br><br>Image analysis: Myotubes were then fixed and stained for myosin heavy chain (MF20 clone, eBioscience, #14-6503-82; used at 1:100) and myotube diameters analyzed by ImageJ. Diaphragm sections were imaged on a Nikon C2 confocal microscope with a 10x objective and the myofiber types and sizes were analyzed in an automated manner with the Nikon Elements software by using the inverse threshold of laminin |

a2 immunostaining to determine myofiber boundaries. The myosin heavy chain staining was used to classify type I (red), type IIA (green), and presumed type IIX/IIB myofibers (black, i.e. that were not stained for MHC I or IIA). After myofibers were classified by type, myofiber size was estimated in an automated manner by the Nikon Elements software via the Feret's minimal diameter, a geometrical parameter for the analysis of unevenly shaped or cut objects179. For the quantification of the number of myofibers, all fibers in the diaphragm cross-sections were counted based on the myofiber borders identified by laminin a2 immunostaining.

For manuscripts utilizing custom algorithms or software that are central to the research but not yet described in published literature, software must be made available to editors and reviewers. We strongly encourage code deposition in a community repository (e.g. GitHub). See the Nature Research guidelines for submitting code & software for further information.

## Data

Policy information about availability of data

All manuscripts must include a data availability statement. This statement should provide the following information, where applicable:

- Accession codes, unique identifiers, or web links for publicly available datasets
- A list of figures that have associated raw data
- A description of any restrictions on data availability

The RNA-seq data discussed in this publication have been deposited in the NCBI's Gene Expression Omnibus and are publicly accessible through GEO Series accession numbers GSE156815, GSE158581, and GSE183833, available at the following hyperlinks:
https://www.ncbi.nlm.nih.gov/geo/query/acc.cgi?acc=GSE156815
https://www.ncbi.nlm.nih.gov/geo/query/acc.cgi?acc=GSE158581
https://www.ncbi.nlm.nih.gov/geo/query/acc.cgi?acc=GSE183833
Additional primary data are available in Supplementary Data 1-5 and in the Source Data File.
Publicly-available datasets that have been interrogated in this study are the following and were used to retrieve RNA-seq data from pediatric cancers (ProteinPaint; https://proteinpaint.stjude.org/) and adult cancers (FireBrowse; http://firebrowse.org/viewGene.html?gene=ITGA2B), and for assessing circulating levels of Fibcd1 (Aging Plasma Proteome dataset; https://twc-stanford.shinyapps.io/aging_plasma_proteome/).

# Field-specific reporting

Please select the one below that is the best fit for your research. If you are not sure, read the appropriate sections before making your selection.

☒ Life sciences ☐ Behavioural & social sciences ☐ Ecological, evolutionary & environmental sciences

For a reference copy of the document with all sections, see nature.com/documents/nr-reporting-summary-flat.pdf

# Life sciences study design

All studies must disclose on these points even when the disclosure is negative.

| | |
|---|---|
| Sample size | Sample size was based on trial experiments and/or previous experiments. |
| Data exclusions | No data points were excluded. |
| Replication | Replication in mouse experiments is not standard given their time- and cost-intensive nature. On this basis, mouse studies were not replicated but they included sufficient numbers to account for biological variability. Moreover, multiple techniques and models were used to validate the same findings. Cell culture experiments were done typically 2-3 times and there were no replication attempts that failed. |
| Randomization | The samples/animals were allocated to groups randomly to reduce the chance of batching effects. |
| Blinding | The result of rFibcd1 treatment was assessed by an investigator blinded to the experimental conditions. Likewise, treadmill experiments were done by a blinded investigator. Image analysis and quantitation was done in an automated (and hence blinded) manner. All experiments were reviewed by 2-3 independent persons. |

# Reporting for specific materials, systems and methods

We require information from authors about some types of materials, experimental systems and methods used in many studies. Here, indicate whether each material, system or method listed is relevant to your study. If you are not sure if a list item applies to your research, read the appropriate section before selecting a response.

## Materials & experimental systems

| n/a | Involved in the study |
|---|---|
| ☐ | ☒ Antibodies |
| ☐ | ☒ Eukaryotic cell lines |
| ☒ | ☐ Palaeontology and archaeology |
| ☐ | ☒ Animals and other organisms |
| ☒ | ☐ Human research participants |
| ☒ | ☐ Clinical data |
| ☒ | ☐ Dual use research of concern |

## Methods

| n/a | Involved in the study |
|---|---|
| ☒ | ☐ ChIP-seq |
| ☒ | ☐ Flow cytometry |
| ☒ | ☐ MRI-based neuroimaging |

# Antibodies

| | |
|---|---|
| Antibodies used | All antibodies were used at 1:150 for immunostaining and at 1:1000 for western blot. Mouse anti-phospho-p44/42 MAPK (Erk1/2; Thr202/Tyr204) (Cell Signaling, CST#9106), rabbit anti-phospho-p38 MAPK (Thr180/Tyr182) (Cell Signaling, CST#4511), rabbit anti-phospho-Smad2 (Ser465/467) (Cell Signaling, CST#8828), rabbit anti-phospho-Akt (Ser473; D9e) (Cell Signaling, CST#4060S), rabbit anti-phospho-SAPK/JNK (Thr183/Tyr185; 81E11) (Cell Signaling, CST#4668S), rabbit anti-a-Tubulin (11h10) (Cell Signaling, CST#2125), rabbit anti-SQSTM1/p62 (Cell Signaling, CST#5114), mouse anti-Flag (M2) (Sigma, #F3165), mouse IgG2b anti-myosin heavy chain type I (DSHB, #BA-F8), mouse IgG1 anti-myosin heavy chain type IIA (DSHB, #SC-71), and rat anti-laminin a2 (4H8-2; Santa Cruz, #sc-59854). For immunostaining, the following secondary antibodies (1:200) were used: anti-mouse IgM Alexa 555 (Life Technologies, A21426), anti-mouse IgG1 Alexa488 (Life Technologies, #A21121), and anti-rat IgG Alexa647 (Life Technologies, #A21247). For western blot, appropriate HRP-conjugated secondary antibodies (Cell Signaling #7074, #7076) were used at 1:2000. |
| Validation | These antibodies were validated for their use in human and mouse cells by the supplier and by us via Western blots and immunostaining on the basis of the correct MW and/or staining pattern. All the antibodies here used have been extensively used in thousands of publications. |

# Eukaryotic cell lines

Policy information about cell lines

| | |
|---|---|
| Cell line source(s) | C2C12 mouse muscle cells (ATCC, #CRL-1772); HEK293T (ATCC, #CRL-3216); MAST360B/SJMEL030083_X2 and MAST552A/SJMEL031086_X1 (Childhood Solid Tumor Network collection at St. Jude Children's Research Hospital); B16F0 and B16F10 melanoma cells (ATCC #CRL-6322 and #CRL-6475); the human MDA-MB-231 breast cancer cells (ATCC #CRM-HTB-26); the mouse Ep5 and Ep5ExTu (Labelle lab collection), E0771 (CH3 BioSystems #94A001), 67NR (F. Miller lab collection, Karmanos Cancer Institute), and 4T1 (ATCC #CRL-2539) breast cancer cells; the human LS180 colorectal adenocarcinoma cells (ATCC #CL-187); the mouse MC38 colorectal adenocarcinoma cells (D. Vignali lab collection, University of Pittsburgh); the human U-2OS-Luc/YFP, 143B-Luc/YFP, and Saos-2 osteosarcoma cells (ATCC #HTB-96, #CRL-8303, and #HTB-85); and the mouse Lewis lung carcinoma cells (LLC) (ATCC, #CRL-1642). |
| Authentication | These cell lines were authenticated via phenotypic, morphological, and RNA-seq/qPCR analyses, which demonstrated a gene expression profile as expected based on previous reports. |
| Mycoplasma contamination | These cell lines tested negative for mycoplasma contamination and were screened regularly to avoid such possibility. |
| Commonly misidentified lines (See ICLAC register) | No commonly misidentified cell lines were used in this study. |

# Animals and other organisms

Policy information about studies involving animals; ARRIVE guidelines recommended for reporting animal research

| | |
|---|---|
| Laboratory animals | Male C57BL/6J (The Jackson Laboratory, JAX#000664) mice were utilized at 4 months of age. Female 2-month-old NCI Ath/nude mice were purchased from Charles River Laboratory. All mice were housed and handled in accordance with approved St. Jude Children's Research Hospital Institutional Animal Care and Use Committee protocols. Mice were housed in a ventilated rodent-housing system with a controlled temperature (22-23C), 40% humidity, 12-hour light/dark cycle, and given free access to food and water. Humane endpoints were not exceeded in any experiment. Drosophila melanogaster strains were housed at 25C, 60% humidity, and a 12-hour light/dark cycle, and are listed in Supplementary Data 1. |
| Wild animals | No wild animals were used in this study. |
| Field-collected samples | No field-collected samples were used in this study. |
| Ethics oversight | All mice were housed in the Animal Resource Center at St. Jude Children's Research Hospital, and handled in accordance with approved St. Jude Children's Research Hospital Institutional Animal Care and Use Committee (IACUC). Additional accreditation of the Animal Resource Center at St. Jude Children's Research Hospital was provided by the Association for Assessment and Accreditation of Laboratory Animal Care (AAALAC). Protocols employed in this study were approved by the Animal Care and Use Committee (Protocol #563).No approval is needed for Drosophila as this is an invertebrate organism. |

Note that full information on the approval of the study protocol must also be provided in the manuscript.
