# Supplementary material for: The myokine Fibcd1 is an endogenous determinant of myofiber size and mitigates cancer-induced myofiber atrophy
Source: Nat Commun. 2022 May 2;13:2370. doi: 10.1038/s41467-022-30120-1 (PMC9061726; doi:10.1038/s41467-022-30120-1)
Supplement: Supplementary file 3 — Description of Additional Supplementary Files [file 41467_2022_30120_MOESM3_ESM.pdf]

Title: Supplementary Data 1.
Description: Drosophila RNAi screening identifies evolutionary conserved myokines that regulate myofiber size.

Title: Supplementary Data 2.
Description: Genes regulated in diaphragm muscles from mice with LLC cancers but not in diaphragm muscles from mice with LLC cancers upon treatment with rFibcd1.

Title: Supplementary Data 3.
Description: qRT-PCR oligos.

Title: Supplementary Data 4.
Description: Genes that are commonly upregulated and downregulated in the diaphragm, tibialis anterior, and soleus muscles of mice with cachectic versus non-cachectic melanomas.

Title: Supplementary Data 5.
Description: Genes differentially regulated in diaphragm muscles of mice with cachectic versus non cachectic melanomas, and treated with either rFibcd1 or mock.
